# Supplementary material for: Kinesin-4 KIF21B is a potent microtubule pausing factor
Source: eLife. 2017 Mar 14;6:e24746. doi: 10.7554/eLife.24746 (PMC5383399; doi:10.7554/eLife.24746)
Supplement: Supplementary file 1. — Samples of purified KIF21B proteins were loaded on SDS-PAGE, isolated from the gel after in-gel digestion and subsequently analyzed by mass spectrometry to test for purity. All identified proteins are included in Supplementary file 1in alphabetical order. Indicated are the molecular weight and the number of unique peptides found for identified proteins in the different KIF21B samples. In total, 121 proteins were identified for KIF21B-FL-GFP, 183 for KIF21B-FL-ΔrCC-GFP, 107 for KIF21B-MD-CCΔrCC-GFP, 92 for GFP-L-WD40 and 63 for KIF21B-MD-CC1-GFP. DOI: http://dx.doi.org/10.7554/eLife.24746.039 [file elife-24746-supp1.docx]

|  |  |  | **# Unique Peptides** | | | | |
| --- | --- | --- | --- | --- | --- | --- | --- |
| **Description** | **Accession** | **MW [kDa]** | **FL-GFP** | **FLdelrCC- GFP** | **MD-CC delrCC-GFP** | **GFP- L-WD40** | **MD-CC1- GFP** |
| 14-3-3 protein beta/alpha | P31946 | 28.1 | 7 | 7 | 7 | 0 | 0 |
| 14-3-3 protein epsilon | P62258 | 29.2 | 19 | 18 | 19 | 0 | 0 |
| 14-3-3 protein eta | Q04917 | 28.2 | 12 | 11 | 11 | 0 | 0 |
| 14-3-3 protein gamma | P61981 | 28.3 | 7 | 7 | 6 | 0 | 0 |
| 14-3-3 protein theta | P27348 | 27.7 | 7 | 4 | 6 | 2 | 2 |
| 14-3-3 protein zeta/delta | P63104 | 27.7 | 11 | 11 | 12 | 0 | 0 |
| 26S proteasome non-ATPase  regulatory subunit 2 | Q13200 | 100.1 | 1 | 1 | 0 | 0 | 0 |
| 60 kDa heat shock protein,  mitochondrial | P10809 | 61.0 | 4 | 2 | 1 | 0 | 0 |
| 60S ribosomal protein L23 | P62829 | 14.9 | 0 | 0 | 0 | 0 | 3 |
| 78 kDa glucose-regulated protein | P11021 | 72.3 | 7 | 8 | 0 | 1 | 0 |
| Actin, alpha cardiac muscle 1 | P68032 | 42.0 | 3 | 4 | 2 | 0 | 0 |
| Actin, cytoplasmic 1 | P60709 | 41.7 | 6 | 7 | 6 | 11 | 4 |
| Actin-related protein 2 | P61160 | 44.7 | 6 | 10 | 0 | 0 | 0 |
| Actin-related protein 2/3  complex subunit 1A | Q92747 | 41.5 | 4 | 7 | 0 | 0 | 0 |
| Actin-related protein 2/3  complex subunit 1B | O15143 | 40.9 | 3 | 7 | 0 | 0 | 0 |
| Actin-related protein 2/3  complex subunit 2 | O15144 | 34.3 | 4 | 7 | 0 | 0 | 0 |
| Actin-related protein 2/3  complex subunit 3 | O15145 | 20.5 | 2 | 3 | 0 | 0 | 0 |
| Actin-related protein 2/3  complex subunit 4 | P59998 | 19.7 | 5 | 5 | 1 | 0 | 0 |
| Actin-related protein 2/3  complex subunit 5 | O15511 | 16.3 | 1 | 1 | 0 | 0 | 0 |
| Actin-related protein 2/3  complex subunit 5-like protein | Q9BPX5 | 16.9 | 2 | 1 | 1 | 0 | 0 |
| Actin-related protein 3 | P61158 | 47.3 | 8 | 10 | 0 | 0 | 0 |
| Adenylate kinase 2, mitochondrial | P54819 | 26.5 | 0 | 1 | 0 | 0 | 0 |
| ADP/ATP translocase 2 | P05141 | 32.8 | 0 | 0 | 2 | 0 | 2 |
| Alpha-actinin-4 | O43707 | 104.8 | 4 | 7 | 0 | 0 | 0 |
| Alpha-adducin | P35611 | 80.9 | 0 | 3 | 0 | 0 | 0 |
| Alpha-enolase | P06733 | 47.1 | 1 | 1 | 2 | 0 | 1 |
| Annexin A2 | P07355 | 38.6 | 1 | 3 | 6 | 3 | 0 |
| AP-1 complex subunit  gamma-like 2 | O75843 | 87.1 | 0 | 1 | 0 | 0 | 0 |
| Apolipoprotein D | P05090 | 21.3 | 0 | 0 | 0 | 1 | 0 |
| Apoptosis-inducing factor 1,  mitochondrial | O95831 | 66.9 | 1 | 4 | 6 | 1 | 0 |
| Arginase-1 | P05089 | 34.7 | 2 | 2 | 4 | 4 | 1 |
| Asparagine synthetase  domain-containing protein 1 | Q9NWL6 | 72.0 | 0 | 0 | 1 | 1 | 0 |
| BAG family molecular  chaperone regulator 2 | O95816 | 23.8 | 0 | 3 | 0 | 4 | 0 |
| Band 4.1-like protein 2 | O43491 | 112.5 | 4 | 11 | 0 | 0 | 0 |
| Band 4.1-like protein 3 | Q9Y2J2 | 120.6 | 0 | 2 | 0 | 0 | 0 |
| Basigin | P35613 | 42.2 | 1 | 0 | 0 | 0 | 0 |
| Beta-actin-like protein 2 | Q562R1 | 42.0 | 1 | 1 | 0 | 1 | 0 |
| Bleomycin hydrolase | Q13867 | 52.5 | 0 | 1 | 1 | 1 | 0 |
| Bromodomain-containing  protein 2 | P25440 | 88.0 | 0 | 1 | 0 | 0 | 0 |
| Cadherin-related family  member 2 | Q9BYE9 | 141.5 | 1 | 0 | 0 | 0 | 1 |
| Calmodulin | P62158 | 16.8 | 3 | 2 | 0 | 0 | 0 |
| Calmodulin-like protein 5 | Q9NZT1 | 15.9 | 0 | 0 | 0 | 1 | 0 |
| Carboxypeptidase A4 | Q9UI42 | 47.3 | 0 | 1 | 0 | 0 | 0 |
| Casein kinase II subunit alpha | P68400 | 45.1 | 3 | 10 | 5 | 0 | 0 |
| Casein kinase II subunit alpha' | P19784 | 41.2 | 0 | 1 | 0 | 0 | 0 |
| Casein kinase II subunit beta | P67870 | 24.9 | 0 | 1 | 0 | 0 | 0 |
| Caspase-14 | P31944 | 27.7 | 0 | 0 | 3 | 1 | 1 |
| Catalase | P04040 | 59.7 | 1 | 0 | 4 | 0 | 0 |
| Cathepsin D | P07339 | 44.5 | 1 | 1 | 1 | 1 | 1 |
| Cilia- and flagella-associated  protein 221 | Q4G0U5 | 96.8 | 0 | 0 | 1 | 0 | 0 |
| Clathrin heavy chain 1 | Q00610 | 191.5 | 4 | 16 | 0 | 0 | 0 |
| Clathrin light chain A | P09496 | 27.1 | 0 | 1 | 0 | 0 | 0 |
| Cofilin-1 | P23528 | 18.5 | 2 | 2 | 0 | 0 | 0 |
| Cofilin-2 | Q9Y281 | 18.7 | 1 | 1 | 0 | 0 | 0 |
| Corneodesmosin | Q15517 | 51.5 | 0 | 1 | 1 | 0 | 1 |
| Cornifin-B | P22528 | 9.9 | 0 | 0 | 2 | 0 | 0 |
| CTTNBP2 N-terminal-like protein | Q9P2B4 | 70.1 | 1 | 1 | 1 | 0 | 0 |
| Cyclin-Y | Q8ND76 | 39.3 | 0 | 1 | 0 | 0 | 0 |
| Cystatin-A | P01040 | 11.0 | 1 | 1 | 1 | 2 | 1 |
| Deleted in malignant brain  tumors 1 protein | Q9UGM3 | 260.6 | 0 | 1 | 1 | 1 | 0 |
| Dermcidin | P81605 | 11.3 | 2 | 3 | 3 | 3 | 3 |
| Desmocollin-1 | Q08554 | 99.9 | 1 | 5 | 4 | 5 | 1 |
| Desmocollin-3 | Q14574 | 99.9 | 0 | 0 | 1 | 0 | 0 |
| Desmoglein-1 | Q02413 | 113.7 | 2 | 7 | 15 | 7 | 9 |
| Desmoplakin | P15924 | 331.6 | 13 | 15 | 30 | 26 | 14 |
| Dihydrolipoyl dehydrogenase,  mitochondrial | P09622 | 54.1 | 1 | 5 | 0 | 0 | 0 |
| Dihydrolipoyllysine-residue  succinyltransferase component  of 2-oxoglutarate dehydrogenase  complex, mitochondrial | P36957 | 48.7 | 0 | 1 | 0 | 0 | 0 |
| DNA damage-binding protein 1 | Q16531 | 126.9 | 2 | 9 | 0 | 0 |  |
| DNA-directed RNA polymerase III  subunit RPC1 | O14802 | 155.5 | 0 | 0 | 0 | 0 | 6 |
| DNA-directed RNA polymerases  I, II, and III subunit RPABC1 | P19388 | 24.5 | 0 | 0 | 0 | 0 | 4 |
| DNA-directed RNA polymerases  I, II, and III subunit RPABC3 | P52434 | 17.1 | 0 | 0 | 0 | 0 | 1 |
| DnaJ homolog subfamily B member 11 | Q9UBS4 | 40.5 | 0 | 1 | 0 | 0 | 0 |
| Drebrin | Q16643 | 71.4 | 0 | 2 | 1 | 0 | 0 |
| E3 ubiquitin-protein ligase CHIP | Q9UNE7 | 34.8 | 1 | 4 | 0 | 0 | 0 |
| Elongation factor 1-alpha 1 | P68104 | 50.1 | 6 | 7 | 4 | 5 | 1 |
| Elongation factor 1-gamma | P26641 | 50.1 | 0 | 1 | 0 | 0 | 0 |
| Elongation factor 2 | P13639 | 95.3 | 1 | 1 | 1 | 1 | 0 |
| Elongation factor Tu, mitochondrial | P49411 | 49.5 | 4 | 5 | 0 | 0 | 0 |
| Endothelial protein C receptor | Q9UNN8 | 26.7 | 0 | 1 | 0 | 0 | 0 |
| Erythrocyte band 7 integral  membrane protein | P27105 | 31.7 | 0 | 2 | 0 | 0 | 0 |
| Ethanolamine-phosphate  phospho-lyase | Q8TBG4 | 55.6 | 1 | 0 | 0 | 1 | 0 |
| Eukaryotic initiation factor 4A-I | P60842 | 46.1 | 0 | 0 | 0 | 1 | 0 |
| Exportin-2 | P55060 | 110.3 | 0 | 0 | 0 | 1 | 0 |
| F-actin-capping protein subunit alpha-1 | P52907 | 32.9 | 2 | 0 | 0 | 0 | 0 |
| F-actin-capping protein subunit alpha-2 | P47755 | 32.9 | 0 | 1 | 1 | 0 | 0 |
| F-actin-capping protein subunit beta | P47756 | 31.3 | 3 | 2 | 2 | 0 | 0 |
| F-box only protein 50 | Q6ZVX7 | 30.8 | 1 | 1 | 2 | 2 | 2 |
| F-box/WD repeat-containing protein 11 | Q9UKB1 | 62.1 | 1 | 3 | 0 | 0 | 0 |
| Filaggrin | P20930 | 434.9 | 1 | 1 | 4 | 0 | 0 |
| Filaggrin-2 | Q5D862 | 247.9 | 2 | 2 | 6 | 4 | 2 |
| Filamin-A | P21333 | 280.6 | 3 | 10 | 0 | 0 | 0 |
| Filamin-B | O75369 | 278.0 | 8 | 18 | 0 | 0 | 0 |
| Filamin-C | Q14315 | 290.8 | 5 | 9 | 0 | 0 | 0 |
| Fructose-bisphosphate aldolase A | P04075 | 39.4 | 0 | 0 | 1 | 0 | 0 |
| Galectin-7 | P47929 | 15.1 | 0 | 0 | 0 | 1 | 0 |
| Gamma-adducin | Q9UEY8 | 79.1 | 0 | 3 | 0 | 0 | 0 |
| Gamma-glutamylcyclotransferase | O75223 | 21.0 | 0 | 0 | 2 | 0 | 0 |
| Gasdermin-A | Q96QA5 | 49.3 | 1 | 1 | 2 | 1 | 1 |
| Gelsolin | P06396 | 85.6 | 13 | 7 | 0 | 0 | 0 |
| Glyceraldehyde-3-phosphate  dehydrogenase | P04406 | 36.0 | 2 | 3 | 4 | 5 | 2 |
| Guanine nucleotide-binding  protein G(i) subunit alpha-2 | P04899 | 40.4 | 0 | 2 | 0 | 0 | 0 |
| Guanine nucleotide-binding protein G(I)/G(S)/G(T)  subunit beta-1 | P62873 | 37.4 | 2 | 1 | 0 | 0 | 0 |
| Guanine nucleotide-binding  protein G(I)/G(S)/G(T)  subunit beta-2 | P62879 | 37.3 | 0 | 1 | 0 | 0 | 0 |
| Guanine nucleotide-binding  protein G(k) subunit alpha | P08754 | 40.5 | 2 | 0 | 0 | 0 | 0 |
| Guanine nucleotide-binding  protein G(s) subunit alpha  isoforms short | P63092 | 45.6 | 1 | 3 | 0 | 0 | 0 |
| Guanine nucleotide-binding  protein subunit alpha-11 | P29992 | 42.1 | 0 | 1 | 0 | 0 | 0 |
| Guanine nucleotide-binding  protein subunit beta-2-like 1 | P63244 | 35.1 | 0 | 0 | 0 | 0 | 1 |
| Heat shock 70 kDa protein 1A | P0DMV8 | 70.0 | 24 | 24 | 20 | 25 | 15 |
| Heat shock 70 kDa protein 1-like | P34931 | 70.3 | 2 | 3 | 1 | 4 | 1 |
| Heat shock 70 kDa protein 4 | P34932 | 94.3 | 3 | 5 | 0 | 0 | 0 |
| Heat shock 70 kDa protein 4L | O95757 | 94.5 | 0 | 2 | 0 | 0 | 0 |
| Heat shock 70 kDa protein 6 | P17066 | 71.0 | 0 | 2 | 0 | 0 | 0 |
| Heat shock cognate 71 kDa protein | P11142 | 70.9 | 27 | 26 | 17 | 30 | 10 |
| Heat shock protein 105 kDa | Q92598 | 96.8 | 0 | 4 | 0 | 3 | 0 |
| Heat shock protein 75 kDa,  mitochondrial | Q12931 | 80.1 | 0 | 0 | 0 | 0 | 1 |
| Heat shock protein beta-1 | P04792 | 22.8 | 0 | 1 | 0 | 1 | 0 |
| Heat shock protein HSP 90-alpha | P07900 | 84.6 | 3 | 9 | 0 | 0 | 0 |
| Heat shock protein HSP 90-beta | P08238 | 83.2 | 6 | 7 | 0 | 1 | 0 |
| Hemoglobin subunit beta | P68871 | 16.0 | 0 | 0 | 2 | 0 | 0 |
| Heterogeneous nuclear  ribonucleoprotein K | P61978 | 50.9 | 0 | 1 | 0 | 0 | 0 |
| Heterogeneous nuclear  ribonucleoprotein M | P52272 | 77.5 | 1 | 3 | 0 | 0 | 0 |
| Histidine ammonia-lyase | P42357 | 72.7 | 0 | 0 | 1 | 0 | 0 |
| Histone H1.2 | P16403 | 21.4 | 0 | 0 | 0 | 2 | 0 |
| Histone H2A.Z | P0C0S5 | 13.5 | 0 | 0 | 0 | 1 | 0 |
| Histone H2B type 1-K | O60814 | 13.9 | 0 | 0 | 0 | 4 | 0 |
| Histone H3.1 | P68431 | 15.4 | 0 | 0 | 0 | 2 | 0 |
| Histone H3.3C | Q6NXT2 | 15.2 | 0 | 0 | 1 | 0 | 0 |
| Histone H4 | P62805 | 11.4 | 1 | 2 | 2 | 4 | 0 |
| Hornerin | Q86YZ3 | 282.2 | 10 | 8 | 13 | 9 | 10 |
| Hsp70-binding protein 1 | Q9NZL4 | 39.4 | 0 | 3 | 0 | 2 | 0 |
| Hyaluronan synthase 1 | Q92839 | 64.8 | 0 | 1 | 0 | 1 | 0 |
| Ig alpha-1 chain C region | P01876 | 37.6 | 0 | 2 | 5 | 3 | 0 |
| Ig alpha-2 chain C region | P01877 | 36.5 | 0 | 0 | 0 | 0 | 1 |
| Ig gamma-1 chain C region | P01857 | 36.1 | 0 | 2 | 0 | 0 | 1 |
| Ig gamma-2 chain C region | P01859 | 35.9 | 1 | 0 | 3 | 1 | 0 |
| Ig lambda-2 chain C regions | P0CG05 | 11.3 | 0 | 1 | 2 | 1 | 0 |
| Interferon kappa | Q9P0W0 | 25.2 | 0 | 1 | 0 | 0 | 0 |
| IST1 homolog | P53990 | 39.7 | 0 | 1 | 0 | 0 | 0 |
| Junction plakoglobin | P14923 | 81.7 | 13 | 14 | 14 | 12 | 8 |
| Kinesin-1 heavy chain | P33176 | 109.6 | 0 | 1 | 0 | 0 | 0 |
| Kinesin heavy chain isoform 5C | O60282 | 109.4 | 1 | 0 | 0 | 0 | 0 |
| Kinesin-like protein KIF21A | Q7Z4S6 | 187.1 | 4 | 2 | 0 | 0 | 0 |
| Kinesin-like protein KIF21B | O75037 | 182.5 | 99 | 82 | 76 | 28 | 51 |
| Lactotransferrin | P02788 | 78.1 | 1 | 3 | 4 | 4 | 2 |
| Leucine-rich repeat-containing  protein 15 | Q8TF66 | 64.3 | 0 | 0 | 0 | 5 | 0 |
| Lipoamide acyltransferase  component of branched- chain alpha-keto acid dehydrogenase  complex, mitochondrial | P11182 | 53.5 | 13 | 19 | 4 | 0 | 0 |
| Lipocalin-1 | P31025 | 19.2 | 0 | 2 | 2 | 3 | 3 |
| Loricrin | P23490 | 25.7 | 0 | 0 | 1 | 0 | 0 |
| Ly6/PLAUR domain-containing  protein 5 | Q6UWN5 | 26.9 | 0 | 0 | 1 | 0 | 0 |
| Lymphokine-activated killer  T-cell-originated protein kinase | Q96KB5 | 36.1 | 0 | 0 | 1 | 0 | 1 |
| Lysosomal protective protein | P10619 | 54.4 | 0 | 1 | 0 | 0 | 0 |
| Lysozyme C | P61626 | 16.5 | 2 | 3 | 5 | 6 | 3 |
| Methylcrotonoyl-CoA  carboxylase beta chain, mitochondrial | Q9HCC0 | 61.3 | 0 | 12 | 0 | 0 | 0 |
| Myosin light polypeptide 6 | P60660 | 16.9 | 0 | 2 | 0 | 0 | 0 |
| Myosin regulatory light chain 12A | P19105 | 19.8 | 0 | 1 | 0 | 0 | 0 |
| Myosin-10 | P35580 | 228.9 | 0 | 2 | 0 | 0 | 0 |
| Myosin-9 | P35579 | 226.4 | 0 | 1 | 0 | 0 | 0 |
| Neuroblast differentiation- associated protein AHNAK | Q09666 | 628.7 | 0 | 0 | 1 | 0 | 0 |
| Neutrophil defensin 1 | P59665 | 10.2 | 0 | 1 | 1 | 1 | 0 |
| Neutrophil elastase | P08246 | 28.5 | 0 | 0 | 1 | 0 | 0 |
| Nucleosome assembly  protein 1-like 1 | P55209 | 45.3 | 0 | 1 | 0 | 0 | 0 |
| p53 and DNA damage- regulated protein 1 | Q9NUG6 | 15.5 | 0 | 0 | 0 | 0 | 1 |
| Peroxiredoxin-1 | Q06830 | 22.1 | 3 | 2 | 2 | 0 | 0 |
| Peroxiredoxin-2 | P32119 | 21.9 | 0 | 1 | 1 | 1 | 2 |
| Phosphatidylinositol 4-kinase alpha | P42356 | 231.2 | 0 | 1 | 0 | 0 | 0 |
| PIH1 domain-containing protein 1 | Q9NWS0 | 32.3 | 0 | 0 | 1 | 0 | 7 |
| Plakophilin-1 | Q13835 | 82.8 | 0 | 1 | 2 | 4 | 1 |
| Plectin | Q15149 | 531.5 | 1 | 2 | 0 | 1 | 0 |
| Polymeric immunoglobulin receptor | P01833 | 83.2 | 1 | 0 | 0 | 0 | 0 |
| Poly(rC)-binding protein 1 | Q15365 | 37.5 | 0 | 0 | 0 | 0 | 2 |
| Polyubiquitin-C | P0CG48 | 77.0 | 5 | 3 | 5 | 4 | 3 |
| POTE ankyrin domain  family member E | Q6S8J3 | 121.3 | 1 | 1 | 1 | 0 | 0 |
| POTE ankyrin domain  family member F | A5A3E0 | 121.4 | 0 | 1 | 0 | 0 | 0 |
| Prefoldin subunit 2 | Q9UHV9 | 16.6 | 0 | 0 | 0 | 0 | 4 |
| Prefoldin subunit 6 | O15212 | 14.6 | 0 | 0 | 0 | 0 | 1 |
| Pre-mRNA-splicing factor  CWC22 homolog | Q9HCG8 | 105.4 | 0 | 1 | 0 | 0 | 0 |
| Probable Xaa-Pro  aminopeptidase 3 | Q9NQH7 | 57.0 | 0 | 0 | 1 | 1 | 0 |
| Prolactin-inducible protein | P12273 | 16.6 | 0 | 4 | 3 | 2 | 2 |
| Proteasome subunit alpha type-1 | P25786 | 29.5 | 0 | 1 | 1 | 0 | 0 |
| Proteasome subunit alpha type-4 | P25789 | 29.5 | 0 | 2 | 0 | 0 | 0 |
| Proteasome subunit alpha type-5 | P28066 | 26.4 | 0 | 1 | 0 | 0 | 0 |
| Proteasome subunit alpha type-6 | P60900 | 27.4 | 1 | 0 | 0 | 0 | 0 |
| Proteasome subunit beta type-5 | P28074 | 28.5 | 1 | 1 | 0 | 0 | 0 |
| Proteasome subunit beta type-6 | P28072 | 25.3 | 2 | 1 | 1 | 0 | 0 |
| Protein BEX5 | Q5H9J7 | 12.6 | 0 | 0 | 0 | 1 | 0 |
| Protein POF1B | Q8WVV4 | 68.0 | 0 | 1 | 1 | 0 | 0 |
| Protein S100-A7 | P31151 | 11.5 | 1 | 1 | 1 | 1 | 1 |
| Protein S100-A8 | P05109 | 10.8 | 4 | 4 | 4 | 3 | 1 |
| Protein S100-A9 | P06702 | 13.2 | 1 | 1 | 1 | 1 | 0 |
| Protein UXT | Q9UBK9 | 18.2 | 0 | 0 | 0 | 0 | 2 |
| Protein XRP2 | O75695 | 39.6 | 0 | 1 | 0 | 0 | 0 |
| Protein-glutamine gamma- glutamyltransferase E | Q08188 | 76.6 | 1 | 2 | 8 | 4 | 0 |
| Protein-glutamine gamma- glutamyltransferase K | P22735 | 89.7 | 1 | 1 | 4 | 2 | 0 |
| Putative annexin A2-like protein | A6NMY6 | 38.6 | 0 | 0 | 0 | 0 | 1 |
| Putative nucleoside  diphosphate kinase | O60361 | 15.5 | 0 | 0 | 0 | 0 | 1 |
| Ras-related protein Rap-2a | P10114 | 20.6 | 0 | 1 | 0 | 0 | 0 |
| RBPJ-interacting and  tubulin-associated protein 1 | Q96K30 | 28.6 | 1 | 1 | 1 | 1 | 0 |
| RNA polymerase  II-associated protein 3 | Q9H6T3 | 75.7 | 0 | 0 | 0 | 0 | 17 |
| RuvB-like 1 | Q9Y265 | 50.2 | 0 | 8 | 18 | 0 | 32 |
| RuvB-like 2 | Q9Y230 | 51.1 | 0 | 6 | 22 | 0 | 38 |
| Semenogelin-1 | P04279 | 52.1 | 0 | 0 | 2 | 6 | 0 |
| Semenogelin-2 | Q02383 | 65.4 | 0 | 0 | 0 | 3 | 0 |
| Serpin A12 | Q8IW75 | 47.1 | 0 | 0 | 1 |  | 0 |
| Serpin B12 | Q96P63 | 46.2 | 3 | 5 | 4 | 3 | 2 |
| Serpin B13 | Q9UIV8 | 44.2 | 0 | 0 | 0 | 1 | 0 |
| Serpin B3 | P29508 | 44.5 | 0 | 3 | 2 | 1 | 0 |
| Serum albumin | P02768 | 69.3 | 7 | 13 | 17 | 8 | 6 |
| Sjoegren syndrome/scleroderma  autoantigen 1 | O60232 | 21.5 | 0 | 0 | 1 | 0 | 3 |
| Skin-specific protein 32 | Q5T750 | 26.2 | 1 | 0 | 0 | 0 | 0 |
| Small proline-rich protein 2E | P22531 | 7.8 | 0 | 0 | 1 | 0 | 0 |
| Sodium/potassium-transporting  ATPase subunit alpha-1 | P05023 | 112.8 | 0 | 2 | 0 | 0 | 0 |
| Solute carrier family 2,  facilitated glucose transporter  member 1 | P11166 | 54.0 | 0 | 2 | 0 | 0 | 0 |
| Spectrin alpha chain,  non-erythrocytic 1 | Q13813 | 284.4 | 29 | 43 | 0 | 0 | 0 |
| Spectrin beta chain,  non-erythrocytic 1 | Q01082 | 274.4 | 20 | 32 | 0 | 0 | 0 |
| Stress-70 protein, mitochondrial | P38646 | 73.6 | 12 | 11 | 1 | 8 | 0 |
| Stress-induced-phosphoprotein 1 | P31948 | 62.6 | 0 | 1 | 0 | 0 | 0 |
| Suprabasin | Q6UWP8 | 60.5 | 1 | 2 | 2 | 0 | 0 |
| Synaptophysin-like protein 1 | Q16563 | 28.5 | 0 | 1 | 1 | 1 | 0 |
| Synaptosomal-associated protein 23 | O00161 | 23.3 | 0 | 1 | 0 | 0 | 0 |
| Target of Myb protein 1 | O60784 | 53.8 | 0 | 1 | 0 | 0 | 0 |
| T-complex protein 1 subunit alpha | P17987 | 60.3 | 12 | 13 | 1 | 15 | 0 |
| T-complex protein 1 subunit beta | P78371 | 57.5 | 16 | 15 | 0 | 15 | 0 |
| T-complex protein 1 subunit delta | P50991 | 57.9 | 16 | 11 | 0 | 14 | 0 |
| T-complex protein 1 subunit epsilon | P48643 | 59.6 | 9 | 7 | 0 | 9 | 0 |
| T-complex protein 1 subunit eta | Q99832 | 59.3 | 15 | 12 | 0 | 14 | 1 |
| T-complex protein 1 subunit gamma | P49368 | 60.5 | 14 | 14 | 0 | 11 | 0 |
| T-complex protein 1 subunit theta | P50990 | 59.6 | 20 | 18 | 0 | 22 | 0 |
| T-complex protein 1 subunit zeta | P40227 | 58.0 | 14 | 10 | 1 | 12 | 0 |
| TGF-beta-activated kinase 1 and  MAP3K7-binding protein 3 | Q8N5C8 | 78.6 | 0 | 1 | 0 | 1 | 0 |
| Thioredoxin | P10599 | 11.7 | 1 | 2 | 3 | 1 | 0 |
| Toll-interacting protein | Q9H0E2 | 30.3 | 0 | 2 | 0 | 0 | 0 |
| Transcription factor MafB | Q9Y5Q3 | 35.8 | 1 | 0 | 0 | 0 | 0 |
| Transcription intermediary  factor 1-beta | Q13263 | 88.5 | 0 | 1 | 0 | 0 | 0 |
| Transcriptional adapter 2-beta | Q86TJ2 | 48.4 | 0 | 1 | 1 | 1 | 0 |
| Translocon-associated  protein subunit delta | P51571 | 19.0 | 0 | 1 | 1 | 0 | 0 |
| Triosephosphate isomerase | P60174 | 30.8 | 1 | 0 | 1 | 0 | 0 |
| Tropomyosin alpha-3 chain | P06753 | 32.9 | 2 | 4 | 0 | 0 | 0 |
| Tubulin alpha-1B chain | P68363 | 50.1 | 0 | 1 | 0 | 0 | 0 |
| Tubulin alpha-1C chain | Q9BQE3 | 49.9 | 5 | 1 | 2 | 4 | 2 |
| Tubulin beta chain | P07437 | 49.6 | 1 | 4 | 2 | 4 | 1 |
| Tubulin beta-2B chain | Q9BVA1 | 49.9 | 1 | 0 | 0 | 0 | 0 |
| Tubulin beta-4B chain | P68371 | 49.8 | 2 | 2 | 0 | 0 | 0 |
| Unconventional myosin-VI | Q9UM54 | 149.6 | 1 | 3 | 0 | 0 | 0 |
| Unconventional prefoldin  RPB5 interactor 1 | O94763 | 59.8 | 0 | 0 | 0 | 0 | 7 |
| UPF0193 protein EVG1 | Q9BZE7 | 24.9 | 0 | 0 | 0 | 1 | 0 |
| Vimentin | P08670 | 53.6 | 15 | 17 | 7 | 13 | 0 |
| V-set and immunoglobulin  domain-containing protein 8 | Q5VU13 | 43.9 | 0 | 0 | 0 | 1 | 0 |
| WD repeat-containing protein 1 | O75083 | 66.2 | 4 | 5 | 0 | 0 | 0 |
| WD repeat-containing protein 5 | P61964 | 36.6 | 0 | 3 | 0 | 0 | 0 |
| WD repeat-containing protein 92 | Q96MX6 | 39.7 | 0 | 0 | 0 | 0 | 10 |
| Zinc-alpha-2-glycoprotein | P25311 | 34.2 | 2 | 2 | 2 | 3 | 1 |
