# Supplementary material for: Kinesin-4 KIF21B is a potent microtubule pausing factor
Source: eLife. 2017 Mar 14;6:e24746. doi: 10.7554/eLife.24746 (PMC5383399; doi:10.7554/eLife.24746)
Supplement: Supplementary file 2. — DOI: http://dx.doi.org/10.7554/eLife.24746.040 [file elife-24746-supp2.docx]

|  | **Sample** | **Lognormal (Best fit values)** | | |
| --- | --- | --- | --- | --- |
|  |  | **A** | **GeoMean** | **GeoSD** |
| **Figure 2A** | GFP | 141.5 ± 0.86 | 1980 ± 9 | 1.78 ± 0.007 |
|  | EB3-GFP | 104.6 ± 1.1 | 4969 ± 54 | 2.1 ± 0.018 |
|  | MD-CC1-GFP | 110 ± 1.1 | 5886 ± 60 | 2.05 ± 0.017 |
| **Figure 2D** | GFP | 2457 ± 11 | 4208 ± 4.3 | 1.36 ± 0.002 |
|  | MD-CC1-GFP | 2665 ± 94 | 7470 ± 92 | 1.33 ± 0.015 |
| **Figure 3 – Figure Supplement 1A** | GFP | 424 ± 6.8 | 7463 ± 70.25 | 1.6 ± 0.014 |
|  | EB3-GFP | 349 ± 4.6 | 11310 ± 108 | 1.74 ± 0.015 |
|  | KIF21B-FL-GFP | 413 ± 4.4 | 10711 ± 71 | 1.62 ± 0.01 |
| **Figure 5C** | GFP | 1434 ± 28 | 5108 ± 66 | 1.74 ± 0.025 |
|  | KIF5B-560 | 1731 ± 82 | 9463 ± 266 | 1.6 ± 0.041 |
| **Figure 5D** | KIF5B-560 | 667 ± 14 | 6160 ± 103 | 1.81 ± 0.025 |
|  | KIF21B-FL-GFP | 655 ± 28 | 5271 ± 169 | 1.77 ± 0.049 |
| **Figure 5E** | KIF21B-FL-GFP running on seed | 1584 ± 15 | 5206 ± 29 | 1.63 ± 0.01 |
|  | KIF21B-FL-GFP running on lattice | 1821 ± 52 | 5534 ± 84 | 1.53 ± 0.02 |
| **Figure 5F** | KIF5B-560 | 783 ± 11 | 5755 ± 54 | 1.68 ± 0.014 |
|  | KIF21B-FL-GFP | 673 ± 40 | 5069 ± 260 | 1.88 ± 0.082 |
| **Figure 5G** | KIF5B-560 | 783 ± 11 | 5755 ± 54 | 1.68 ± 0.014 |
|  | KIF21B-FL-GFP | 737 ± 25 | 5477 ± 133 | 1.73 ± 0.037 |
| **Figure 5H** | KIF5B-560 | 763 ± 14 | 6129 ± 73 | 1.66 ± 0.018 |
|  | KIF21B-FL-GFP | 737 ± 17 | 4637 ± 78 | 1.75 ± 0.025 |
| **Figure 5– Figure Supplement 1A** | KIF21B-FL-GFP running on seed | 1378 ± 34 | 6613 ± 116 | 1.74 ± 0.028 |
|  | KIF21B-FL-GFP running on lattice | 1723 ± 100 | 6618 ± 234 | 1.6 ± 0.049 |
| **Figure 5 – Figure Supplement 1B** | KIF21B-FL-GFP running on seed | 673 ± 17 | 5557 ± 107 | 1.77 ± 0.03 |
|  | KIF21B-FL-GFP running on lattice | 873 ± 73 | 4988 ± 260 | 1.63 ± 0.07 |
| **Figure 5 – Figure Supplement 1C** | KIF21B-FL-GFP running on seed | 718 ± 18 | 5146 ± 85 | 1.67 ± 0.025 |
|  | KIF21B-FL-GFP running on lattice | 729 ± 18 | 5720 ± 98 | 1.72 ± 0.026 |
| **Figure 7A** | GFP | 125 ± 0.7 | 1689 ± 8.7 | 1.93 ± 0.008 |
|  | EB3-GFP | 99 ± 0.8 | 5797 ± 54.3 | 2.21 ± 0.016 |
|  | FL-∆rCC-GFP | 94 ± 0.7 | 5364 ± 49.1 | 2.31 ± 0.016 |
|  | MD-CC∆rCC-GFP | 89 ± 1.6 | 6000 ± 156 | 2.28 ± 0.039 |
